# Relevance of Objective Measures in Psychiatric Disorders—Rest-Activity Rhythm and Psychophysiological Measures

**DOI:** 10.1007/s11920-021-01291-3

**Published:** 2021-10-29

**Authors:** Eunsoo Moon, Michelle Yang, Quinta Seon, Outi Linnaranta

**Affiliations:** 1 Department of Psychiatry, Pusan National University School of Medicine, Yangsan, Republic of Korea; 2 Department of Psychiatry and Biomedical Institute, Pusan National University Hospital, Busan, Republic of Korea; 3 Interdisciplinary Health Sciences, University of Ottawa, Ottawa, ON Canada; 4 Department of Psychiatry, McGill University, Montreal, QC Canada; 5 Mental Health Unit, Finnish Institute for Health and Welfare, P.O. Box 30, 00271 Helsinki, Finland; 6 Douglas Centre for Sleep and Biological Rhythms, Douglas Mental Health University Institute, 6875 LaSalle Boulevard, Montreal, QC H4H 1R3 Canada

**Keywords:** Rest-activity rhythm, Psychophysiological, Heart rate variability, Skin conductance, Objective measurement

## Abstract

**Purpose of Review:**

We present a review of recent methods of objective measurement in psychiatry and psychology with a focus on home monitoring and its utility in guiding treatment.

**Recent Findings:**

For individualized diagnostics and treatment of insomnia, actigraphy can generate clinically useful graphical presentations of sleep timing and patterns. Psychophysiological measures may complement psychometrics by tracking parallel changes in physiological responses and emotional functioning, especially during therapy for trauma symptoms and emotion regulation. It seems that rather than defining universal cut-offs, an individualised range of variability could characterize treatment response.

**Summary:**

Wearable actigraphy and psychophysiological sensors are promising devices to provide biofeedback and guide treatment. Use of feasible and reliable technology during experimental and clinical procedures may necessitate defining healthy and abnormal responses in different populations and pathological states. We present a “call for action” towards further collaborative work to enable large scale use of objective measures.

## Introduction

**Fig. 1:**
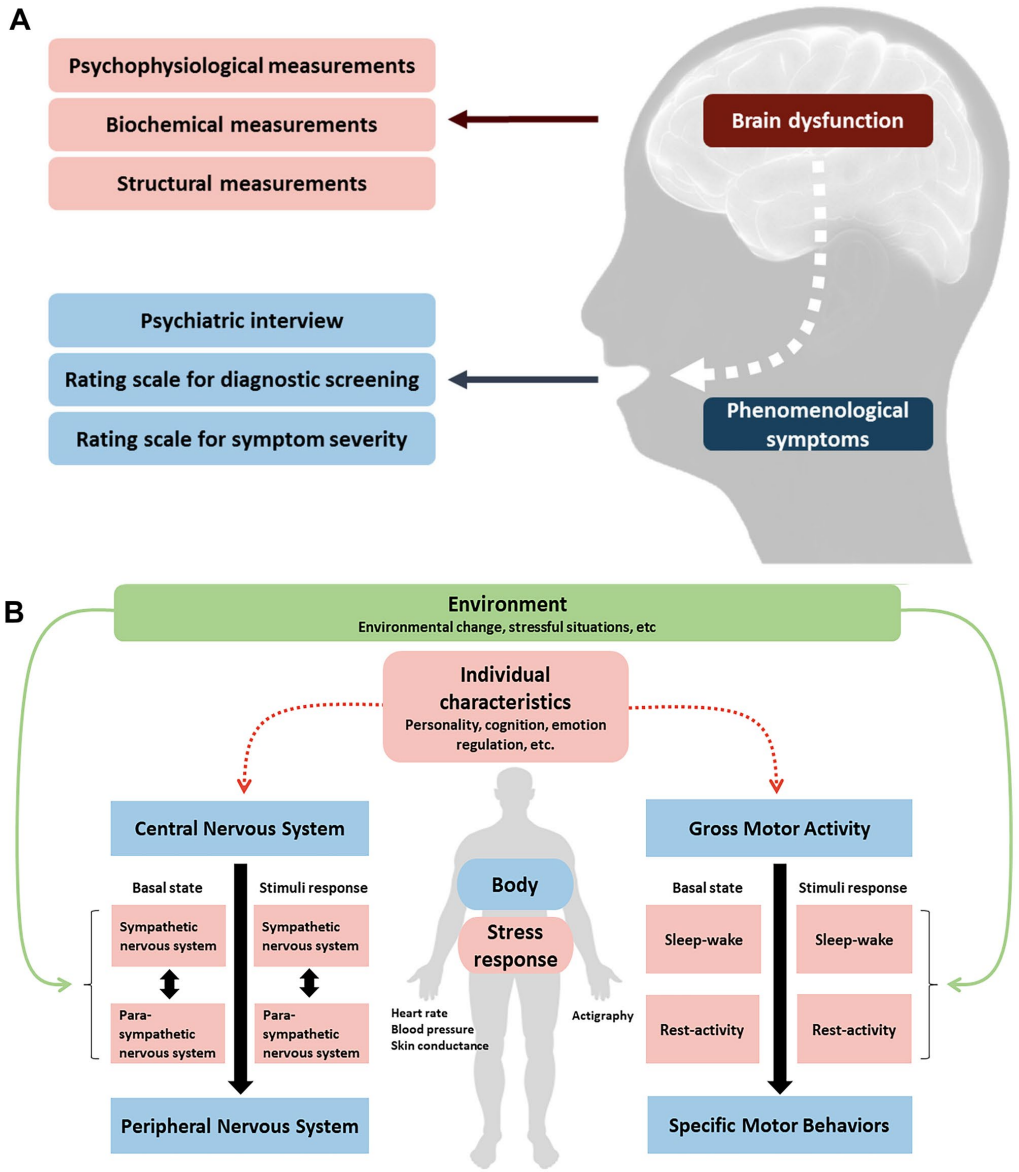
**A** and **B** Various measurements of brain dysfunction related to psychiatric disorders (**A**) and psychophysiological measurements on interactions between brain activity, peripheral responses, behaviors, and environments (**B**)

The Central Nervous System (CNS) controls biological, cognitive, and emotional states. Any of these states can be directly reflected in phenomenological psychiatric symptoms. For instance, motor activity, sleep, and interday variability in daily rhythms may indirectly indicate CNS function, and be measured by a wrist actigraphy. Equally, regulating emotional states is robustly linked to physiological functions regulated by the CNS. Accordingly, psychophysiological signals can reflect the effects of emotional states on brain activity, interactions between CNS and peripheral nervous system, that cause the brain signals, as well as interactions between the body and environment that provoke psychophysiological responses (Fig. 1). Interactions between CNS and peripheral nervous system can be assessed by responses of the autonomic nervous system (ANS) to laboratory standards or everyday triggers. Since the sympathetic nervous system (SNS) and parasympathetic nervous system (PNS) are each dominant under different conditions, optimal or abnormal levels of various markers of the SNS/PNS can provide insight into individual characteristics of stress responses.

To date, clinical interview, structured diagnostic interviews, and psychometric scales have been the standard of psychiatric assessment. Since the availability of trained personnel limits the use of structured interviews and interviewer-rated symptom scales, subjective surveys have been considered the most feasible format of evaluation. However, several factors limit the reliability of traditional psychometrics. Firstly, the time to recognize and label an emotion (such as current anxiety level) through a subjective report can confound the accuracy of the label as well as the individual’s own appraisal of the emotional experience [1]. Secondly, it is common to encounter recall bias when administering psychometric tests [2]. Thirdly, the role that cultural and linguistic factors play on written emotion and symptom questionnaire remains limiting [3]. Response discordance also limits value of subjective reports; for example, subjective insomnia has not correlated with objective actigraphy data consistently [4]. Most importantly, scores where fluctuating states are averaged over time do not correspond to the real-life, naturalistic psychological experience.

As such, developing objective measurements of these biological states to complement psychometric evaluation of symptoms may be of interest. Here, we integrate the current knowledge on the utility of objective measurement for clinical use, such as for diagnostics, outcome measurement, or targeting and planning psychotherapeutic interventions. We will evaluate the information on utility of device data for clinicians as well as reliability of the data.

## State-of-the-Art Literature Review

### Rest-Activity Rhythm

Traditionally, polysomnography (PSG) or electroencephalography (ECG) is the gold standard for measuring quality of sleep. PSG is optimal for the diagnosis for sleep apnea and is necessary for a detailed analysis of sleep quality [5]. Using PSG, sleep apnea has been diagnosed in up to 60% of a general psychiatric outpatient population [6]. In addition, individuals with obstructive sleep apnea diagnosed by PSG have a pooled prevalence of 35% and 32% of depressive or anxious symptoms [7]. The high prevalence of sleep apnea and considerably higher complaints of sleep problems and fatigue in the psychiatric population than the general population, and the need for a specialized laboratory and costs for personnel limits clinical utility of PSG [8].

Actigraphy, a wearable watch-like device, can detect activity and inactivity, most commonly wrist movements. Accumulating data shows that actigraphy can be a convenient device for clinical estimation of individual characteristics of sleep [9•, 10, 11••]. This includes estimation of normality of the sleep–wake cycle, such as sleep phasing (regularity, advance, or delay), and correspondence of sleep periods to the actual clock time (Table 1). A minimum of 14 days of recording is recommended for a pattern analysis [11••]. Actigraphy has been validated against PSG [12], also in all major groups with a severe mental illness, and the prevalence of sleep problems has been objectively confirmed to be increased among patients with schizophrenia, bipolar disorder, attention-deficit hyperactivity disorder, and autism spectrum disorder [13–16] (Table 1). Actigraphy characterizes well circadian rhythms and circadian rhythm disorders [11••, 17–19]. Methods to describe total sleep time, sleep onset time, and sleep offset time have been shown not to be reliable in a psychiatric population, where fragmented sleep is common, and accordingly, parameters calculated based on these values, such as sleep efficiency or sleep fragmentation index, might be less accurate. As such, actigraphy remains the most feasible, low-cost method for reliable home monitoring of sleep and circadian rhythm sleep–wake disorders [11••].

**Table 1:** Overview of findings in actigraphy studies in the field of psychiatry

	**Dementia**	**SCHZ**	**BD**	**MDD**	**ASD**	**ADHD**	**ED**	**PTSD**
**Motor activity**	Daytime activity ↓ [82]	Total activity ↓ [83]	Total activity ↓ [83] Variability of motor activity↑ [9•] Less robust rhythm of motor activity in mania [84] Total activity ↓ in depression [85]	Total activity ↓ [86]	Nighttime movements ↑ [87] Night-to-night variability ↑ [87]	Total activity ↑ [88, 89]	Daily physical activity ↓ in BED [90]	
**Nonparametric analysis**	RA↓[82] IV↑[82] IS↓[82]	RA ↔ [83] IV ↔ [83] IS ↔ [83]	RA ↔ [83] IV ↔ [83] IS ↔ [83]	RA ↔ [91] IV ↔ [91] IS ↔ [91]		RA ↔ [92] IV↓ [92] IS↑ [92]	IV ↔ in BED [93] IS↑ in BED [93]	RA↓ [94] IV ↔ [94] IS↓ [94]
**CR analysis**	Amplitude ↓ [82]	Disrupted [83]	Disrupted [83] Delayed [22]	MESOR ↓[22] Delayed (in antidepressant use) [22]	Delayed [22]	Delayed in adult ADHD [22]	Delayed [95] MESOR ↓ in BED [90, 93] Amplitude ↓ in BED [90, 93]	
**Graphical observation**		Non-24-h-rhythms [96]	Non-24-h-rhythms Irregular phasing [10]		Delayed [97]	Delayed in adult ADHD [98]	Irregular eating patterns [95]	

*SCHZ* schizophrenia, *BD* bipolar disorder, *MDD* major depressive disorder, *ASD* autism spectrum disorder, *ADHD* attention-deficit hyperactivity disorder, *ED* eating disorder, *BED* binge eating disorder, *PTSD* post-traumatic stress disorder, *CR* circadian rhythm, *RA* relative amplitude, *IS* inter-daily stability, *IV* intra-daily variability, *MESOR* midline estimating statistic of rhythm Symbols: **↑** increased; **↓** decreased; ↔ unchanged

Usability and accuracy of actigraphy data among psychiatric patients, with a high prevalence of sleep–wake dysregulation and fragmented sleep, have improved remarkably over recent years. Recent work has validated open-access algorithms for three-dimensional inactivity data [20, 21] and to produce inactograms for graphical observation of sleep [22]. Two novel parameters, Center of Daily Inactivity (CenDI) and Consolidation of Sleep (ConDI), were recently presented by our team, and describe stability, phasing, and period of sleep using these algorithms [22]. The parameters detect circadian abnormalities with clinically relevant correlations, such as with insomnia and depressive symptoms (Table 1).

### Psychophysiological Measures

Individual differences in emotional processing and emotion regulation are reflected in sympathetic and parasympathetic functions of the ANS. Accordingly, the ANS response to both internal and external events, such as stress or experimental stimuli, can elucidate the mechanistic role of different pathological phenotypes, which could improve primary prevention efforts, and timing and targeting of treatments. These emotional processes have been experimentally phenotyped by physiological reactions in real-time using wearable sensors [23, 24, 25••, 26•]. Resting levels and reactivity to stimuli are the most prominent phenotypic metrics in evaluating longitudinal changes and predicting response to therapy [27, 28•, 29] (Table 2). The most clinically relevant parameters at rest include heart rate (HR), heart-rate variability (HRV), respiratory sinus arrhythmia (RSA), and Diastolic blood pressure (DBP). Electrodermal activity (EDA), electromyography (EMG) startle response, and eye movement and pupillometry facial EMG are clinically relevant for reactivity to stressors.

**Table 2:** Overview of psychophysiological measures related to autonomic nervous system in the field of psychiatry

	**Basal state**	**Stimuli response**
**Reflecting state**	Chronic or longitudinal state	Acute or responsive state
**Optimal psychophysiological metrics**	Resting heart rate (HR) [99, 100] Interbeat interval (IBI) [101] Heart rate variability (HRV) [99] Respiratory sinus arrhythmia (RSA) at baseline [41] Skin conductance level (SCL) [100, 101]	Heart rate reactivity (HR) [29] High-frequency heart rate variability reactivity (HF-HRV) [73, 100] Systolic/diastolic blood pressure (S/D BP) [29] Skin conductance response (SCR) [101] Fear-potential startle (FPS) [61] Electromyography (EMG) startle response [99, 100] Respiratory sinus arrhythmia reactivity (RSA withdrawal) [73, 102]
**Interpretation**	Physiological restoration/recovery [103] Tonic level component [104] Increased vagal regulation [105] Parasympathetic (cholinergic) nervous system (PNS) arousal > sympathetic (noradrenergic) nervous system arousal (SNS) [106] Elevevated parasympathetic tone [107]	Physiological mobilisation [103] Phasic level component [104] Reduced vagal regulation [105] Sympathetic (noradrenergic) nervous system (SNS) arousal > parasympathetic (cholinergic) nervous system (PNS) arousal [106] Elevated sympathetic activation [107]
**Findings in psychiatric disorders**	Resting HR↑ in PTSD [29] Resting HR ↓ in MDD [62] HRV↓ in MDD, GAD, BD, PDA, SCHZ, PTSD [29, 101, 107–109] IBI↓ in MDD [101] SCL ↓in MDD [101] RSA↓ in MDD, PTSD, SCHZ [41, 99, 110]	HR reactivity↑in PTSD, specific phobia, BPD [29, 50, 111] HR reactivity↓in MDD [62] HF-HRV↓in MDD [62] SCR↑in specific phobia, GAD, BPD, PTSD [29, 111, 112] SCR↓in MDD, BD [113] EMG startle response↑in PTSD [29] EMG startle response↓in MDD, PTSD with dissociative symptoms [113, 114] BP ↑ in PTSD [29] BP↓in MDD [115] SBP↓in MDD, GAD [116] DBP↑in MDD, PTSD [29, 116] FPS ↓in MDD [61] FPS↑in SAD, PDA, Specific phobia, GAD, PTSD [61] RSA withdrawal↑ in PTSD, GAD, comorbid MDD, and anxiety/PTSD, SAD, PDA [102]

*HRV* heart rate variability, *RSA* respiratory sinus arrhythmia, *HR* heart rate, *BP* blood pressure, *SBP* systolic blood pressure, *DBP* dystolic blood pressure, *IBI* interbeat interval, *SCR* skin conductance response, *SCL* skin conductance level, *FPS* fear-potential startle, *SNS* sympathetic nervous system, *PNS* parasympathetic nervous system, *MDD* major depressive disorder, *PTSD* post-traumatic stress disorder, *SCHZ* schizophrenia, *GAD* generalized anxiety disorder, *BD* bipolar disorder, *PDA* panic disorder with or without agoraphobia, *SAD* social anxiety disorder, *BPD* borderline personality disorder

### Biofeedback

A common means of treatment that is driven by psychophysiological measurement is biofeedback, where physiological response patterns are presented in real-time [30, 31••, 32]. In HRV biofeedback, the goal is that patients learn to control physiological correlates of originally maladaptive emotional responses. Researchers hypothesize that greater amplitude HRV promotes autonomic homeostasis and resilience; this affects patients’ ability to self-regulate emotions, improve skills in stress management and self-regulatory resource mobilization [31••, 33–36]. Biofeedback to increase High-Frequency (HF)-HRV (0.15–0.4 Hz) is useful for clinical purposes, since this parameter is associated with better emotional regulation [29].

The mechanism of HRV biofeedback is that it encourages a resonance between respiratory and baroflex rhythms to increase HRV amplitude, which can subsequently decrease depressive, anxiety, anger, trauma, and insomnia symptoms [37, 38]. This can be done through resonance frequency training, where biofeedback trains slow paced breathing, pacing breath rhythms at six breaths per minute [38]. During HRV biofeedback, to determine the resonant frequency of the patient, and subsequently, to track improvements in levels of respiratory sinus arrythmia through their resonant frequency training, devices such as respirometers are needed. Current development allows such devices to be used both in vivo in clinics, home settings, and mobile settings (ambulatory tracking). This device is a flexible sensor band that detects waveforms during resonance frequency training and indicates if the desired respiration rate is followed [39, 40].

Inactograms could be used as objective biofeedback, providing more detailed information than the widely used mood charts. We presented a rapid, clinician-friendly algorithm for this purpose previously [22].

### Stress Response and Reactivity

In experimental paradigms that include emotion provoking stimuli or stress paradigms, patients differ in their response as compared to healthy controls. Patients can exhibit heightened reactivity to startling sounds and trauma cues, and delayed recovery from cue-activated startle have been observed transdiagnostically [33, 41, 42] (Table 2). Abnormal expressions in physiological measures have also been linked with an elevated risk of depression, altered cognitive flexibility in patients with PTSD [43], and trouble managing rumination (perseverative cognition) [44], but also with level of resilience [45]. These biomarkers may have utility in conducting large-scale cross-sectional studies for early identification of individuals at risk for developing PTSD and depression, evaluating outcomes of resilience training programs [46], and for identifying pre-clinical forms of psychiatric disorders [47].

### Response to Treatment

The psychophysiological markers have value in predicting high and low responders to psychotherapy or pharmacotherapy [48, 49]. Patients with anxiety, especially phobia, that had the strongest physiological reactions to distressing hierarchy cues, had better responses to systemic desensitization [50]. A higher degree of physiological arousal during pre-treatment or initial sessions predicted larger decreases during therapy, even at longitudinal follow-up [29, 51•, 52]. In particular, immediate physiological responses at baseline, such as EMG, HR, and SC reactivity, have accounted for a significant variance in the longitudinal changes in trauma symptoms [29, 51•, 52].

The value of observing phasic stress responses is in the potential for brief physiological assessment [53•]. In a recent systematic review, improvement in autonomic functioning correlated with a psychometric response to trauma treatment [29]. Increases in resting HRV, and decreases in resting HR and SBP, as well as reactivity to stimuli or imagery as measured by EDA and EMG across treatment trials of PTSD, uniformly indicated treatment response [29]. In some trials, baseline pre-treatment psychophysiological responses predicted higher likelihood of treatment response. Thus, psychophysiological measures have potential utility in the assessment of symptom severity or outcome in PTSD.

### Clinically Meaningful Response

For use as a diagnostic or predictive tool clinically, validated cut-offs for unhealthy and healthy/recovery are necessary. For sleep, it has been proposed that variation of + / − 1 h in the sleep midpoint is normal when selecting healthy controls for research [54]. No guidelines for other sleep parameters are available. For psychophysiological measurement, cut-offs should objectively discriminate between maladaptive and adaptive autonomic responses to stimuli and emotion. For example, higher than average fear and anxiety expressions have characterized some psychiatric disorders; these are recognized in the National Institutes of Health Research Domain Criteria as constituting separate negative valence system constructs [50]. As well, interpreting HRV levels within the scope of population values has predictive utility and can indicate self-regulation [55]. Thus, cut-off levels of biomarkers may have potential in identifying patterns associated with different disorders [56]. However, an issue which precludes the validity of psychophysiological cut-offs is that normative values to use as cut-offs are currently not defined, nor standard conditions for stimuli and measurement for comparability. Certain reference normative values that indicate adaptive ANS functioning during rest have been proposed to be 120/80 mmHg for BP, ≤ 5 microsiemens for SCL, and ≤ 3 µV for EMG with a wide bandpass [57]. However, several factors affect these values, such as age, gender, and fitness [58, 59].

Alternatively, individual responses could be considered when evaluating longitudinal changes and assessing psychiatric presentations [39]. In one sample, intraindividual variability in sleep rather than absolute findings was of importance for seeking help [60]. In a sample of patients with a bipolar disorder, subjective insomnia correlated with lower sleep consolidation and later timing of sleep, but not variability [22]. For psychophysiological data, given that the testing conditions remain the same across time per individual, treatment response can be reflected in individual responses during rest or reactivity to laboratory stressors through non-invasive measurements with high discriminative power [47, 61]. Since some measures, such as HRV and BP, vary over time between-subjects and it may be more appropriate to compare participant’s physiological levels in reference to their individual mean across multiple laboratory visits [55]. Through individual-level comparisons, we could demonstrate degrees of association of emotional distress and individual physiological responses.

Another example of intraindividual change is the use of difference in means, a physiological metric whereby individual differences in mean BPM, microsiemens, or other physiological units are calculated across treatment points or between conditions (i.e. stressful condition vs neutral condition) [48, 62, 63]. This metric has been associated with the degree of clinician-rated prognosis, is proposed to reflect symptom dimensions or subclassification within PTSD, and even points towards profiles of symptoms with distinct psychophysiologies [48, 64].

Other studies which use individual responses to guide treatment have used within-session-change (WSC) and between-session change (BSC) of physiological metrics. BSC and WSC have been positively associated with treatment outcome, and may even predict treatment outcome of psychoeducation, imaginal exposure, in vivo exposure, EMDR, and relaxation [65]. Individual physiological arousal patterns have related to components of intervention. This can be done through hierarchical linear modelling—as opposed to traditional difference score approach—a more sensitive test of individual changes in arousal [48, 65].

More support for the use of individual responses comes from research on individual differences in vagal reactivity [55]. In adults, individual differences in vagal flexibility, which influences HRV, HR, and HR acceleration [66], have been less studied than the use of resting levels to psychiatric phenotypes. However, this method of interpreting individual differences in psychophysiology has successfully shown that greater vagally mediated HRV (vmHRV) reactivity is adaptive, major depression is associated with smaller degreases in vmHRV in response to threat, and suicidal ideation is associated to larger decreases in vmHRV in response to sad stimuli [55].

Population-ranged response may be biased because individuals differ pre-to-post intervention (within-subject random variability) in study outcome(s), though not necessarily in treatment response [67]. Atkinsons et al. propose a clinically meaningful definition of magnitude of response, which is a potential solution to the lack of cut-off values. A meaningful response is anchored to changes in the risk of morbidity or another endpoint that matters significantly.

For use in psychiatry, the healthy values would depend on the condition under treatment and its prognosis. Accordingly, rather than to have a transdiagnostically valid cut-off, there is a need for validation of clinically relevant magnitudes of response with clearly defined target groups and aims, such as a clinically meaningful magnitude of PTSD treatment response as measured by a physiological metric.

### Data Processing and Interpretation

For clinical use, sleep parameters or psychophysiological measures need to be clinically interpretable (Table 3). Graphical observation of inactograms does not necessitate any pre-processing: inactograms provide a quick understanding whether the patient has a delay in sleep phase, fragmented sleep, or an abnormal length of the sleep period. Actigraphy data needs to be cleaned from non-wear if dimensional values for average over a certain time are used.

**Table 3:** A call for action towards further collaborative work aiming at suitable devices and tools to enable large scale use of objective measures in clinical psychiatry and psychology

	**User experience**	**Clinical feasibility and utility**	**Accuracy and reliability**
**Actigraphy and sensors**	Portable Inexpensive Durable Can be sterilized Light to wear	Rapid conversion for quality observation, biofeedback, and rapid diagnostics Cut-offs for healthy range Allows tagging certain stressors, eating times, substance use etc. for therapeutic use Long enough monitoring to describe real life values	Graphical presentation is sufficient if can detect individual change Reliable numeric values necessary if dimensional measures are used to define content of treatment or recovery Secure storage of data, including an ID for the patient, accurate timing of data collection in the data, and a secure back-end server Colours coding healthy vs unhealthy values
**Actigraphy**	Breathing materials to avoid itching, waterproof Shows time for those who normally use a watch, to destigmatize use Different models for men and women for destigmatizing use User biofeedback an option	Allows tagging subjective sleep-time and wake-up to define sleep onset latency	Includes heart rate to increase accuracy for sleep Charging allows use for minimum 14 days to detect patterns
**Psychophysiological sensors**	Ambulatory sensor systems, such as those with integrated Bluetooth technology to allow movement during exposure and real-life conditions Non-invasive	Robust to motion artifacts, automatized reliable cleansing of sensor data for biofeedback Allows tagging certain stressors, substance use etc	Charging allows use for minimum 1 h at a time, preferably over 2 weeks

For psychophysiological data, there should be further development that allows for rapid automated data collection and data cleansing [68], with devices and tools that can be clinically implemented. Additionally, clinicians need platforms to effectively transform data into visuals, graphs, synthesized text, or other summaries in real or rapid timing. For diagnostic information or measurement of treatment response, it is necessary to know how the clinician should summarize the session results in medical files.

A challenge for larger scale use would be if all devices necessitate their own user interface to guide clinicians. Equally, instating institute-wide integration with medical files could be an expensive challenge. General validated algorithms, as compared to protected, producer-provided values for parameters and cleansing procedures could guarantee reliability. This would also confirm comparability independent of the device and producer. Security of real-time storage of data is necessary, including an ID for the patient, accurate timing of data collection in the data, and sometimes, a secure back-end server (Table 3).

### Choice of Device

Any device that is targeted to clinical use has to fulfill certain demands. They need to be cleaned according to hospital standards; thus, the materials cannot be sensitive to disinfectants or ultraviolet light. For home monitoring purposes, the devices should have a reasonably long usage time before recharging (Table 3). As an example, most actigraphy devices are charged on a daily basis, while a minimum recommended recording is 2 weeks. Relying on patient’s adherence to device maintenance or data storage is not always realistic or ideal for continuous monitoring. Durability is also a major characteristic necessary in long-term monitoring at home, and device components should be provided for replacements. Thus, using sensors for diagnostic and psychiatric purposes requires further collaboration between device producers and researchers to validate response cut-offs, multimodal assessments, and novel statistical methods to provide a standard of quality of psychophysiological measurement tools.

Three-dimensional actigraphic measurement and devices providing access to the raw data are the reliable devices for research [9•, 10]. While actiwatches and algorithms have reached a robust level of reliability, feasibility for users necessitates further work [9•, 10] (Table 3).

Increasing the use and clinical applications of psychophysiological measurements necessitates digital systems that are portable, inexpensive, and robust to motion artifacts [53•, 69–71] (Table 3). For clinical use, ambulatory sensor systems with integrated Bluetooth capability are more feasible than stable wire transmission devices. However, while they mitigate error from wire data transmission, ambulatory systems are more susceptible to motion artifacts [72•]. Therefore, to increase reliability of use, ambulatory sensor devices should be validated against stationary physiological acquisition systems, and programs which add artifact correction should be integrated [73].

Artifacts are a major barrier for the integration of sensor devices since they contaminate the data. The use of electrodes as a method of capturing data is thought to be the most reliable way for sensors to capture data from the body. In comparison, other methods, including the use of sensor belts, wrist watches, or PPG (photoplethysmography), seem to provide less robust readings [74, 75].

### Appropriate Testing Settings

Actigraphy devices are reliable for home monitoring and have shown value in describing characteristics of real-life sleep. In contrast, psychophysiological recording must be done in a controlled setting, such as having a quiet room and comfortable temperature (Table 3). Optimally, technicians would be present to manage psychophysiological tracking. This is not always feasible even at the clinic, and even less so at home. However, the crucial work of technical personnel is not always guaranteed or standardized across clinical practices such as during psychotherapy, and their presence may have confounding effects on emotions and interaction, reflected in psychophysiology [76]. Finally, for comparability of findings, the stimuli should be standardized. For home monitoring, the future work should seek very robust recordings and validate reactions to every-day life stimuli and normal range of reactivity to those stressors.

## Discussion

### Actigraphy and Psychophysiological Sensors Can Be Clinically Useful

In this review, we illustrate how devices and algorithms are mature for clinical use of actigraphy as a measure for sleep and sleep patterns. Potentially, individual inactograms could help clinicians in providing psychoeducation, or to decide the optimal, personalized timing of administering medication based on the chronotherapeutic knowledge. Furthermore, graphical presentations of psychophysiological reactivity could help clinicians personalize treatment: decisions on appropriate stimulus in therapy or evaluating individual response.

Equally, the integration of biofeedback as part of psychotherapy seems promising. Presenting HR or HRV to the patient during treatment practices is beneficial to psychotherapy. While monitoring respiration is a suitable option for self-management, psychophysiological sensors necessitate further mechanical development to ensure their reliability under clinical conditions, where movement during therapy is necessary and standardized conditions are practically impossible. Overall, many current parameters lack directions about healthy and abnormal levels and variability, which would be necessary for personalized treatment and to define clinical recovery.

### Novel Patient-Oriented and Ecologically Valid Options

The market for commercial devices to monitor sleep, stress, and behavior has rapidly expanded. We must learn a lesson from the consumer interest and integrate objective measures and biofeedback with clinical treatment. Increasing the autonomy of patients, such as allowing them to actively monitor their own symptoms, has shown success in regard to their treatment compliance and treatment outcomes. When objective evaluation is brought to a home environment, the advantages include an ability to evaluate responses naturalistically. We can evaluate sleep and stress responses with real life stimuli, which is needed to confirm ecological validity [26•, 53•]. Experimental research has widely used objective paradigms in research on emotion regulation. The field provides not only clinical applications for illness but also a resilience-orientation.

Conventional at-clinic treatment could be complemented and, at times, replaced, by home monitoring, psychoeducation and self-management. Objective outcome measures such as biofeedback could improve patient participation, evaluation, and development of more efficacious treatments. Real-time monitoring means clinicians can observe causes of psychophysiological responses or conditions, such as insomnia, for therapeutic purposes. Real-time identification of certain behavioral patterns can enable just-in-time adaptive interventions, where e.g. methods of cognitive psychotherapy can be automatized to be provided in a mobile application based on the individual responses or symptoms.

Commercial devices can be low cost, wireless, and provide users with categorical and visual interpretations of physiology. However, in their validation against gold-standard measures, their reliability is not optimal. Thus, feasibility and reliability need to reach a balance “good enough” for ambulatory clinical use.

### Future Health Technology

Both augmented reality and virtual reality (VR) are easily implemented methods standardizing therapeutic environments [77]. Digital technology is advantageous for remote testing and longitudinal follow-ups in psychiatry [77, 78]. Rates of attrition are similar between VR and in vivo interventions; however, remote VR may be the optimal choice for patients with limited access to clinics [79, 80].

Currently, development of VR interventions guided by psychophysiological measures are in initial phases, with fewer options for self-management interventions [28•, 81]. Therapy complemented with VR means increased clinical control and potentially personalized dosing of exposure, as guided by the real-time responses. More development in this area requires interdisciplinary collaboration. It also requires creation of different environments and tools for various groups, such as for populations who are most susceptible to feeling apprehensive and anxious about testing procedures.

### Home-Based Assessments

In the near future, treatment settings that use longitudinal psychophysiological measurement are expected to expand for clinical or home environments [9•, 10]. Clinical trials will soon involve novel testing and sensor systems.

Looking to the future, we could greatly reduce the burden of clinical treatment and research studies on participants with health technologies. For instance, we can expect that recruitment, screening, consent and assignment/randomization through computer algorithms from home or automated incorporation of individual physiological signals into screening and assignment will improve current processes of random assignment in clinical studies. For one, we would have more detailed information on the individual at baseline, including sleep phasing or physiological states. Any of this information could be useful in clinical trials which explore personalized treatments, be it for inclusion, selecting type of treatment or dosing of medication, or defining response. In addition, while traditional clinical trials focus on symptom changes as a primary outcome, new paradigms may be developed to analyze multi-dimensional effects on physiological signal changes with continuous psychophysiological tracking or sleep monitoring. This strategy can also minimize risk of bias in subjective reports and facilitate the analysis of various effects of treatment across medical conditions.

### Applications to Neuroscience

In comparison to other assessment tools, real-time monitoring more clearly demonstrates temporal and longitudinal changes in clinical outcome. Therefore, it is a promising way to monitor changes in brain activity and interactions between brain activity and psychophysiology (e.g., brain and body). Equally, we can study physiological associations with neurochemical changes, hormonal changes, and the hypothalamic–pituitary–adrenal axis. There is potential to deepen the understanding on the etiology and pathophysiology of psychiatric disorders with real-time assessment.

## Limitations

This is a narrative, state-of-the-art review with a focus on the most recent literature. No systematic search was done.

## Conclusions

Accumulating technological advancements and increasing accuracy of measurement allow us to conclude that objective measurement is a promising avenue in clinical psychiatry. A “call for action” towards further collaborative work aiming at suitable devices and tools is necessary. This work is essential before large scale use of objective measures in clinical psychiatry and psychology is possible. Ideally, participatory development includes patients, clinicians, clinical researchers, experts in implementation science, and industrial partners. The clinical development includes validation of characteristics for healthy values and for treatment response and potentially, qualitative evaluation to confirm the sustainability and acceptability of devices by patients themselves. 
